# *In vivo* magnetic resonance imaging of the interstitial pressure gradients (pgMRI) using a pulsatile poroelastic computational model

**DOI:** 10.1098/rsfs.2024.0044

**Published:** 2025-04-04

**Authors:** Matthew McGarry, Damian Sowinski, Likun Tan, John Weaver, Jacobus J. M. Zwanenburg, Keith Paulsen

**Affiliations:** ^1^ Thayer School of Engineering, Dartmouth College, Hanover, NH 03755, USA; ^2^ Department of Physics and Astronomy, University of Rochester, Rochester, NY 14627, USA; ^3^ Dartmouth-Hitchcock Medical Center, Lebanon, NH 03756, USA; ^4^ Translational Neuroimaging Group, Center for Image Sciences, University Medical Center Utrecht, Utrecht, The Netherlands

**Keywords:** glymphatic system, brain pulsatility, poroelasticity, interstitial fluid flow, pressure gradient imaging

## Abstract

Fluid movement in the interstitial space of the brain affects the clearance of waste products, which is an important factor in the pathophysiology of dementia. Estimating interstitial fluid (ISF) flow is critical to understanding these processes; yet, it has proven difficult to measure non-invasively. The pulsatile component of ISF flow may be particularly important for clearance, e.g. by facilitating fluid mixing. Directly measuring ISF flows is challenging due to the slow velocities and small volume fractions involved; however, pulsatile flows present a unique opportunity as their driving forces can be estimated from observations of pulsatile tissue motion. In this work, we present pressure gradient magnetic resonance imaging (pgMRI), which assimilates retrospectively gated pulsatile tissue deformations measured with a displacement encoding with stimulated echoes MRI sequence into a patient-specific poroelastic computational model by estimating the distribution of fluid sources. The new method is demonstrated to recover a spherical fluid source accurately from synthetic data with simulated noise of up to 20%, and to produce not previously reported *in vivo* brain fluid source images along with companion images of the three-dimensional stresses and pressure gradients which drive ISF movement. Repeated exams of four healthy volunteers demonstrated variability below 10% for pgMRI parameters in most cases.

## 1. Introduction

The movement of fluids through brain tissue is vital as it carries the required resources for tissue function (including therapeutic agents) and clears away waste products. Imaging fluid movement and pressure is important for studying a range of diseases, as well as evaluating normal brain function. Conditions such as stroke and cerebral artery diseases affect the movement of blood, and the recently proposed glymphatic hypothesis suggests that disruption in the movement of interstitial fluid (ISF) during sleep is a causative pathway for neurodegenerative conditions such as Alzheimer’s disease [1–3]. The ability to study ISF flow in the human brain is a crucial first step towards the development of effective interventions with next-generation therapeutic agents [4,5]. A particular challenge in studying ISF is the extremely slow velocities involved, which have contributed to some of the controversy in the literature about glymphatic function.

Magnetic resonance imaging (MRI) has been used to measure the movement of neurofluids with varying degrees of success. Observations of the movement of injected contrast agents over time suggest that ISF velocities are orders of magnitude smaller than those of blood, making direct imaging of glymphatic flows in a single session very challenging [6]. The difficulties in measuring fluid flow directly in the brain render any properties associated with flow valuable to our understanding. The flow, $\vec{q}$, of fluids in porous media such as ISF is generated from pressure gradients, ∇P, through Darcy’s law, $\vec{q}=\kappa \nabla P$, where the hydraulic conductivity, κ, parameterizes the ease with which the fluid flows.

The static component of the ISF pressure gradient created by the continuous turnover of fluid provides few viable avenues for non-invasive measurement [7]. The pulsatile component of ∇P is produced by the tissue stresses and blood pressure gradients generated as blood is forced into the deformable brain with each heartbeat [8,9]. Two-photon microscopy measurements of particle movements in mouse brain [10] suggest that pulsatility plays a major role in glymphatic transport by not only enhancing diffusive movement through advection but also directly contributing to bulk flow through peristalsis generated by changes in hydraulic resistance across the cardiac cycle [11–14]. A simple analogy is a dirty sponge under running water—repeatedly squeezing the sponge clears the contaminants more quickly than constant flow. Pulsatile pressure gradients provide a unique opportunity for measurement as the cyclic motion resulting from pulsatile pressure changes is measurable *in vivo* with MRI sequences and can be exploited to gain information on the driving forces and hydraulic properties which govern ISF flow. Insights into these factors will improve our understanding of fluid flow in the human brain.

**Figure 1.**
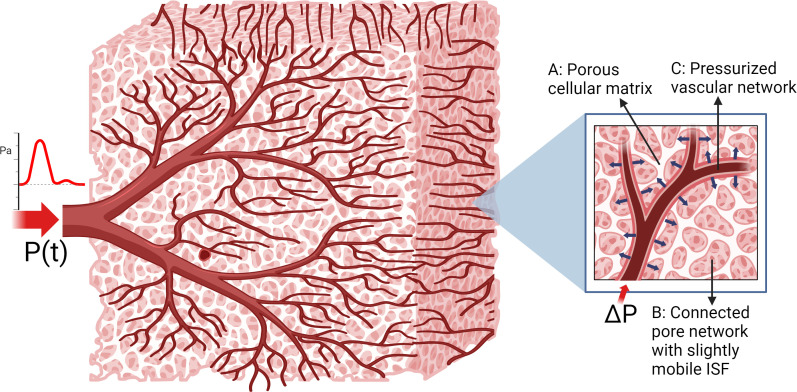
Diagram of the assumed poroelastic brain tissue structure and mechanism of pulsatile motion. A network of blood vessels carries pressurized fluid (C) throughout the brain, and pressure changes within individual vessels push on the surrounding tissues as their walls expand. This movement causes stresses and pressure gradients in tissue (A,B) which drive the pulsatile motion of ISF.

Rather than attempting to measure extremely slow glymphatic flows directly, we estimate the distribution of forces which generate pressure gradients in the ISF and drive glymphatic flow. Here, pulsatile displacements of brain tissue are measured using retrospectively gated motion-sensitized MRI sequences. At cardiac frequencies, brain tissue can be described as a fluid-saturated porous matrix where the hydromechanical behaviour is governed by the equations of poroelasticity, which is a continuum model consisting of a porous elastic solid with one or more compartments of infiltrating pore fluid. The network of blood vessels is driven by the heart and provides the primary source of pressure-induced pulsation. The porous cellular matrix containing low mobility ISF is compressed as the vessels deform, and the stresses generated combined with the blood pressure gradients drive the very small-scale pulsatile motion of the ISF as illustrated in figure 1.

**Figure 2.**
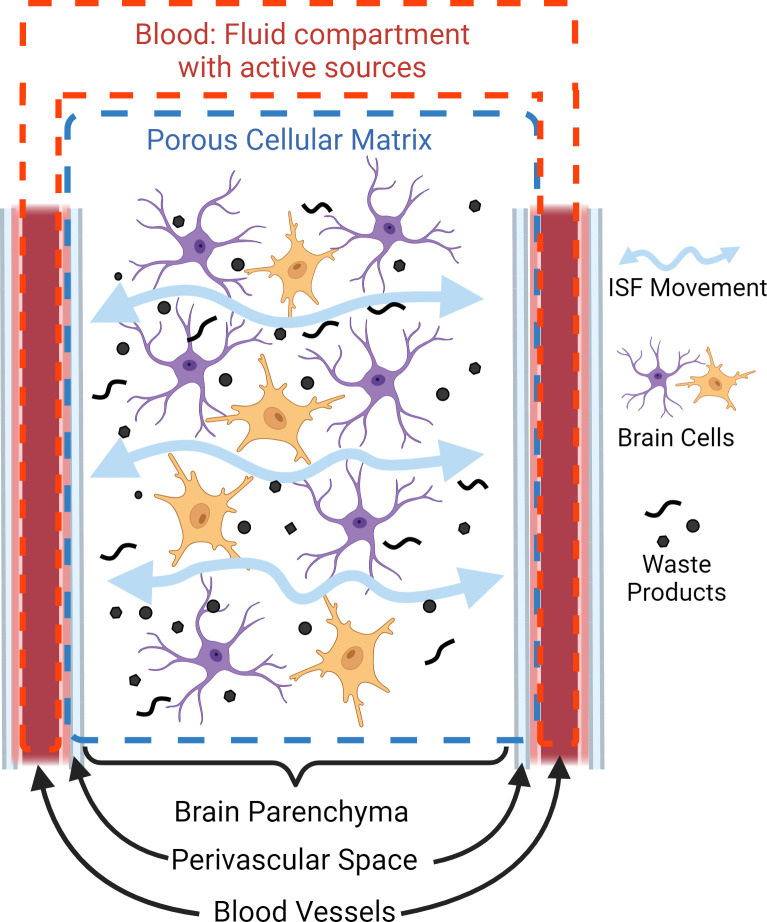
Partitioning of brain tissue components into poroelastic compartments. We assume that the volume of perivascular fluids is small compared with the ISF volume and lump these in a single-porous cellular matrix compartment. The stresses generated in this compartment provide the driving forces for these passive fluids, where their motion is much slower than the active fluid compartment which is directly driven by the cardiac cycle.

Figure 2 is a simplified representation of components of brain tissue and the glymphatic system, and indicates how each is included in our single-compartment poroelastic model with active fluid sources. The network of blood-filled vessels fills the role of the infiltrating pore fluid, with active pressure sources that drive pulsatile motion. Since the mobility of the ISF is much lower than that of blood [15], we assume that redistribution of ISF has a negligible effect on the pulsatile dynamics of the system over the cardiac cycle; hence, we combine the ISF and solid cellular components into the porous solid matrix. We can then compute stresses in the porous matrix and blood pressure gradients, which provide the driving forces for the pulsatile ISF movement (which is very small compared with the blood and solid tissue motions).

In this work, we assimilate MRI measurements into a poroelastic computational model by estimating a distribution of pulsatile driving sources in the blood which reproduce the measured tissue pulsation fields. Once this patient-specific model is generated, we compute the otherwise unmeasurable tissue stresses and blood pressure gradients which drive pulsatile ISF motion. These maps can be produced non-invasively in a clinically acceptable imaging time. They are likely to be informative for our understanding of the role of pulsatile ISF movement in the glymphatic system, and eventually, the technique has the potential for diagnosis and monitoring of brain diseases, especially those involved in disruptions in fluid transport. Our technique is also amenable to extensions into multi-fluid systems that directly estimate ISF flow using multicompartment poroelastic models.

In this article, we present the details of our methods and show examples of novel *in vivo* pulsatile fluid source images and concomitant distributions of associated tissue stresses and pressure in four healthy volunteers with repeated measurements. Future work will refine the models and computational techniques further, and test the method in a larger population of subjects with interventions designed to alter the neurofluid environment in predictable ways to explore the potential clinical value of these new image surrogates of ISF flow.

## 2. Methods

Accurate modelling of fluid transport in a full poroelastic brain model is difficult; even the most advanced methods can only match available *in vivo* measurements approximately [16]. Rather than attempting to model the arterial system and boundary conditions (BCs) precisely to predict full brain response, we instead assimilate MRI-measured pulsatile displacement measurements into a computational model by estimating how much blood volume pulsatility needs to be present across the brain to reproduce the observed pulsatile displacements in a poroelastic finite element (FE) approximation.

### 2.1. Pulsatile displacement measurements

Measurements of the pulsatile motion of brain tissue with retrospectively gated phase-contrast MRI sequences with strong motion encoding gradients have been used in intrinsic actuation MR elastography (IA-MRE) to image the elastic properties of brain tissue [17–19], as well as the hydraulic conductivity, which describes the ease of fluid flow through the tissue under a pressure gradient [20,21]. Imaging time and signal-to-noise ratio (SNR) constraints limited the imaged volume with these sequences to 16 slices around the ventricles. Poroelastic modelling requires BCs to be defined for the fluid and solid components. The brain surface has obvious BC choices of zero fluid flow through the pia mater and solid displacements at the boundary provided by MRI; however, BCs for the top and bottom slices of a limited imaging volume are more difficult to define. Recently, displacement encoding with stimulated echoes (DENSE) sequences has been demonstrated, which allows whole-brain imaging of sub-millimetre pulsatile motion with good SNR in a clinically acceptable imaging time [22–25], and avoids modelling errors from inappropriate BCs in most of the brain. Retrospective gating allows faster data acquisition and reduces imaging artefacts.

### 2.2. Poroelastic model with fluid source

Each frequency component of the response is analysed separately by assuming steady-state time-harmonic behaviour for successive cardiac cycles, so that the displacement is $\vec{u}\left(\vec{x},t\right)=Re\{\vec{U}\left(\vec{x}\right)e^{i\omega t}\}$, pressure is $p\left(\vec{x},t\right)=Re\left\{P\left(\vec{x}\right)e^{i\omega t}\right\}$ and forcing is $\vec{f}\left(\vec{x},t\right)=Re\{\vec{F}\left(\vec{x}\right)e^{i\omega t}\}$, where uppercase letters denote complex-valued amplitudes, which encode the amplitude and phase of the harmonic behaviour at cardiac frequency component ω (in rad s^−1^) for each location, $\vec{x}$. Poroelastic motion of brain tissue with a single-fluid compartment at radial frequency ω is described by [26,27]


(2.1*a*) $$\nabla \cdot \mu \left(\nabla \vec{U}+\nabla {\vec{U}}^{T}\right)+\nabla \left(\frac{\mu \nu }{1-2\nu }\right)\nabla \cdot \vec{U}-\left(1-\beta \right)\nabla P=-\left(\rho -\beta \rho_{f}\right)\omega^{2}\vec{U}+\vec{F},$$

(2.1*b*) $$\nabla \cdot \left(\beta \nabla P\right)+\rho_{f}\omega^{2}\nabla \left(\left(1-\beta \right)\vec{U}\right)=i\omega \rho_{f}\gamma ,$$

(2.1*c*) $$\beta =\frac{\omega \phi^{2}\rho_{f}\kappa }{\omega \kappa \left(\rho_{a}+\phi \rho_{f}\right)+i\phi^{2}}.$$

Here, the tissue response is represented by the displacement of the solid matrix, $\vec{U}$, and pore fluid pressure field, P. Elastic properties of the solid matrix are shear modulus, μ, and Poisson ratio, ν. Note that these quantities are the properties of the drained porous matrix, so near-incompressibility and ν→0.5 are not expected as in single-phase tissue models. Fluid-related parameters include the porosity, ϕ, hydraulic conductivity [28], κ, and three densities: $\rho ,\;\rho_{f}$ and $\rho_{a}$, which are the solid, fluid and apparent mass densities, respectively. The right-hand side of the pressure equation (2.1*b*) describes the forcing term of the fluid through the parameter, γ, which is an internal fluid source with units s^−1^. Positive values inject fluid into the pore spaces, whereas negative values remove it. Under assumptions of steady-state pulsatile components, the net change in fluid content over the cardiac cycle is zero; thus, we are only modelling the pulsatile component at frequency ω.

The poroelastic governing partial differential equations in equation (2.1*a*) are discretized in a FE system with N nodes:


(2.2) [K({θ})]{U}={F}.

The bolded notation used here denotes vectors and matrices constructed from the degrees of freedom of spatially distributed points in the computational representation as {v} and [M], respectively, whereas vector field quantities such as displacement in equation (2.1*a*) are represented as $\vec{U}$. The 4N×4N stiffness matrix, [K], contains the discretized elastic and fluid related parameters represented as {θ}. The response vector $\left\{U\right\}={\left\{U_{x}^{\left(1\right)}\;U_{y}^{\left(1\right)}\;U_{z}^{\left(1\right)}\;P^{\left(1\right)}\;u_{x}^{\left(2\right)}\;u_{y}^{\left(2\right)}\;u_{z}^{\left(2\right)}\;P^{\left(2\right)}\cdots U_{z}^{\left(N\right)}\;P^{\left(N\right)}\right\}}^{T}$ contains the displacement and pressure at each of N FE nodes.

### 2.3. Pressure gradient magnetic resonance imaging algorithm

Poroelastic MR elastography estimates $\left\{\theta \right\}$ in equation (2.2) through a subzone-based nonlinear inversion (NLI) scheme, and can provide maps of the elastic properties of the tissue [29–31], μ and ν, as well as the hydraulic conductivity [28], κ. Alternatively, known values from the literature can be prescribed for {θ}.

Once $\left\{\theta \right\}$ is known we can recover estimates of terms in the forcing vector, {F}, by using generalized least squares (GLS) to minimize [32]


(2.3) $$\Phi_{GLS}={\left\{\tilde{F}\right\}}^{T}\left[W_{f}\right]\left\{\tilde{F}\right\}+{\left\{{\tilde{U}}_{c}-{\tilde{U}}_{m}\right\}}^{T}\left[W_{\delta }\right]\left\{{\tilde{U}}_{c}-{\tilde{U}}_{m}\right\}.$$

Here, $\left\{\tilde{F}\right\}$, $\left\{{\tilde{U}}_{c}\right\}$ and $\left\{{\tilde{U}}_{m}\right\}$ are ‘demeaned’ vectors representing the perturbations due to unknown forcing, modelling errors and data noise and are constructed by subtracting the known ‘average’ components of forcing, $\left\{\bar{F}\right\}$, and displacement response, $\left\{{\bar{U}}_{c}\right\}$, so that $\left\{\tilde{F}\right\}=\left\{F\right\}-\left\{\bar{F}\right\}$ and $\left\{{\tilde{U}}_{c}\right\}=\left\{U_{c}\right\}-\left\{{\bar{U}}_{c}\right\}$, $\left\{{\tilde{U}}_{m}\right\}=\left\{U_{m}\right\}-\left\{{\bar{U}}_{c}\right\}$. We assume that terms in the measured and computed displacement vectors occur at the same locations, which are ensured by interpolating the measurements onto a computational FE mesh. Alternatively, computed values could be sampled at the measurement points.

$\left[W_{f}\right]$ and $\left[W_{\delta }\right]$ are weighting matrices for the forcing terms and model–data mismatch, respectively. In simplest form, they are diagonal matrices, which normalize the two GLS terms. Using $W_{f}\left(i,i\right)=1/E\left[\tilde{F}{\left(i\right)}^{2}\right]$ encodes the average size of the expected forcing ($E\left[q\right]$ denotes the expected value of a variable q) and selects which terms in $\left\{\tilde{F}\right\}$ are recovered. The fluid source terms, γ,of interest appear at locations of internal nodes of the fluid equations in the FE system of equation (2.2), whereas boundary nodes incorporate the fluid flux BCs. Using $W_{\delta }\left(i,i\right)=1/E\left[{\left({\tilde{U}}_{c}\left(i\right)-{\tilde{U}}_{m}\left(i\right)\right)}^{2}\right]$ allows noise estimates for each measurement to be included by the algorithm. More advanced strategies can be constructed from estimates of the prior inverse covariance of $\left\{\tilde{F}\right\}$ and $\left\{\delta \right\}=\left\{{\tilde{U}}_{c}-{\tilde{U}}_{m}\right\}$ to incorporate expected spatial covariance of the fluid sources [33] or measurement noise [34].

Compared with NLI MRE, which estimates {θ} with a similar minimization approach, estimation of $\left\{\tilde{F}\right\}$ is linear where direct solution can be computed in a single step with methods such as unit responses; however, we opted to use a more memory-efficient iterative solution based on conjugate gradients.

Once $\left\{\tilde{F}\right\}$ has been estimated, the displacement and pressure fields can be computed. These patient-specific poroelastic response fields can then be used to estimate otherwise unmeasurable quantities, such as the solid tissue stresses and blood pressure gradients which drive ISF flow [35]. The six independent components of the symmetric rank 2 stress tensor, represented in Voight notation, are given by


(2.4) $$\left\{\begin{array}{l} S_{xx}\\ S_{yy}\\ S_{zz}\\ S_{xy}\\ S_{xz}\\ S_{yz} \end{array}\right\}=\left[\begin{array}{cccccc} \lambda +2\mu & \lambda & \lambda & 0 & 0 & 0\\ \lambda & \lambda +2\mu & \lambda & 0 & 0 & 0\\ \lambda & \lambda & \lambda +2\mu & 0 & 0 & 0\\ 0 & 0 & 0 & \mu & 0 & 0\\ 0 & 0 & 0 & 0 & \mu & 0\\ 0 & 0 & 0 & 0 & 0 & \mu \end{array}\right]\left\{\begin{array}{c} \epsilon_{xx}\\ \epsilon_{yy}\\ \epsilon_{zz}\\ \epsilon_{xy}\\ \epsilon_{xz}\\ \epsilon_{yz} \end{array}\right\},$$

where λ=$\frac{\mu \nu }{1-2\nu }$ and the strain tensor components are given by $\epsilon_{ij}=\frac{1}{2}\left(\frac{\partial U_{i}}{\partial x_{j}}+\frac{\partial U_{j}}{\partial x_{i}}\right)$. The blood pressure gradients are simply the spatial derivatives of the pressure field, ∂P/∂x, ∂P/∂y and ∂P/∂z.

### 2.4. Simulated data

Implementation of the pgMRI algorithm was confirmed with simulated data. A 90 mm cubic mesh of linear tetrahedral FEs was created and a 20 mm diameter spherical fluid source in the bottom corner of the cube at [15, 15, 15] mm was defined with a fixed value of 10^−4^ s^−1^. Fixed zero-displacement BCs with zero flow were applied to the bottom surface, and stress-free and zero-pressure conditions were enforced on other surfaces. Homogeneous properties of μ=3.3 kPa, λ=10 kPa, $\left\{\rho ,\;\rho_{f},\rho_{a}\right\}=\left\{1000,\;1000,\;150\right\}$ kg m^−3^, ϕ=0.2, $\kappa =10^{-8}$ m^3^ s kg^−1^ were prescribed and a driving frequency of 1 Hz was used. Simulated measurement noise was then added to the computed displacements (Gaussian with standard deviation equal to a percentage of the mean displacements). Simulated displacement fields were then supplied to the pgMRI algorithm to recover maps of the fluid source, which were compared with the prescribed ground truth distributions to determine accuracy.

### 2.5. *In vivo* demonstration

MRI exams with DENSE pulsatile motion measurements of four healthy subjects were considered [24]. Three DENSE scans with displacement encoding in the FH, RL and AP directions were performed to capture the full volume, three-dimensional displacement field across the full cardiac cycle. Parameters for DENSE (ordered foot–head/right–left/anterior–posterior (FH/RL/AP) where appropriate) included displacement encoding gradient, DENC = 0.35/0.175/0.175 mm, repetition time (TR) = 35/30/35 ms, echo time (TE) = 8.7 ms, field of view (FOV) = 250 × 250 × 190 mm^3^, voxel size = 1.95 × 1.95 × 2.2 mm^3^, and 20 retrospectively gated cardiac phases. Complex-valued harmonic motion amplitudes were computed by Fourier transforming across the 20 phase offsets. Scanning was performed on a Philips 7 T MRI system (Philips Healthcare) with 32 channel head coil (Nova Medical), and synchronized to the cardiac cycle with a pulse oximeter attached to the left index finger. The total scan time for the three encoding directions was heartrate dependent, approximately 7 min 12 s at 60 beats min^−1^. Each subject participated in two repeated scans after repositioning in the scanner. Written informed consent was obtained prior to the original imaging study [24], and participants agreed to re-use of their data in other studies such as the one performed here.

The pgMRI algorithm for *in vivo* data assumed approximate homogeneous properties [36,37] with μ=500 Pa, λ=1000 Pa, ϕ=0.2, $\kappa =10^{-7}$ kg m^−3^, and the three poroelastic densities were $\left\{\rho ,\;\rho_{f},\rho_{a}\right\}=\left\{1000,\;1000,\;150\right\}$. Estimates of poroelastic properties are rare in the literature as they represent the properties of the drained solid, whereas most mechanical testing reports single-phase approximations of biphasic tissues [38]. Prior work has shown that the fluid source maps recovered with GLS do not vary substantially with changes in assumed properties [32]. The full brain was meshed with 3 mm tetrahedral elements, giving approximately 45 000 total nodes. Displacement BCs were prescribed from the measured data. Fluid BCs were zero flow on the exterior surface of the brain and zero pressure at the brainstem.

## 3. Results

### 3.1. Simulated data

**Figure 3.**
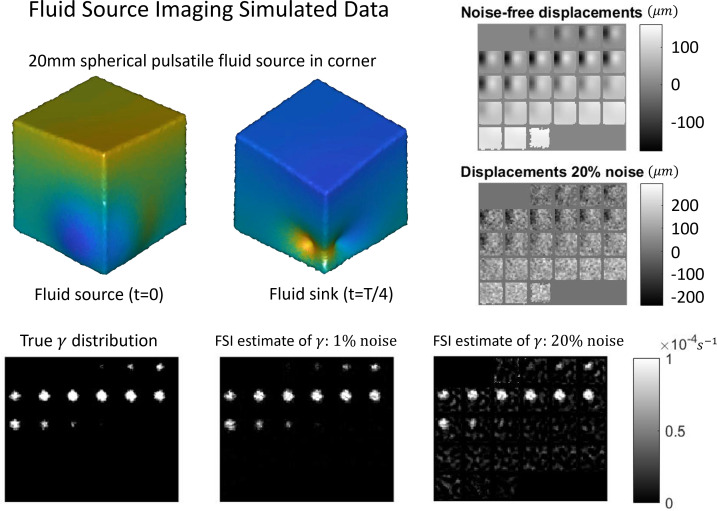
Simulated data demonstrating quantitatively accurate recovery of a spherical fluid source for pulsatile motions. Upper left: Three-dimensional rendering of two points in the pulsatile cycle showing local generation of motions from the fluid source which displaces the rest of the cube through stress propagation (motion is geometrically magnified, colour scale relative to maximum displacement). Top right: Slice montage of one motion component, before and after the addition of 20% simulated measurement noise. Bottom row: Slice montages of the true fluid source compared with the recovered distributions for the noise-free and noise-added cases. Results are stable even with significant measurement noise.

Figure 3 shows the results of a fluid source in an idealized cube simulation. The location of a prescribed spherical fluid source was accurately recovered even with very high levels of simulated measurement noise.

### 3.2. *In vivo* demonstration

**Figure 4.**
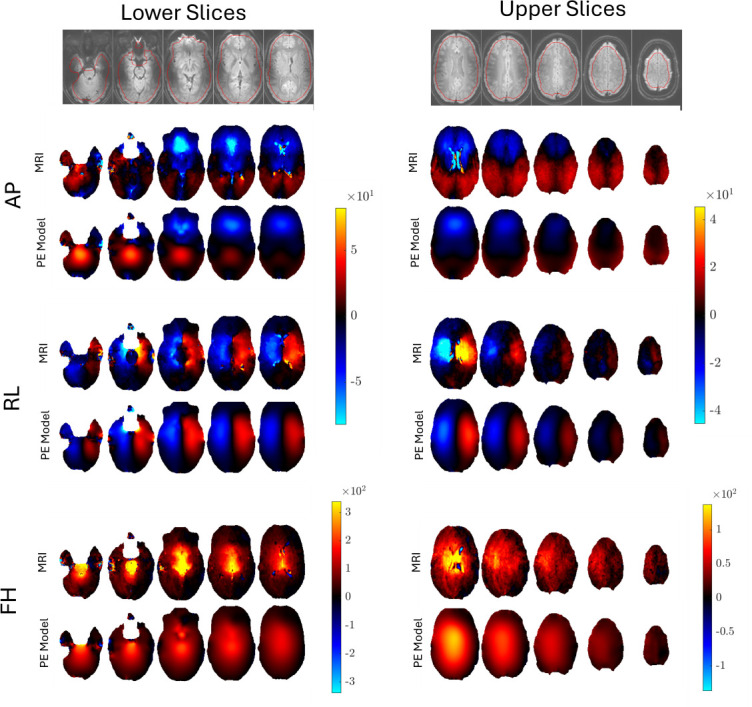
Example MRI measured displacements compared with their computed counterparts from a pulsatile poroelastic (PE) model generated by estimating a distributed pore fluid source from the measured MRI data. These correspond to $U_{m}$ and $U_{c}$, respectively, in equation (2.3). Images/data were acquired during repeat 1 of subject 1. The displayed slices are 9.3 mm apart, and time-harmonic displacement fields near peak systole are shown. Units of μm.

**Figure 5.**
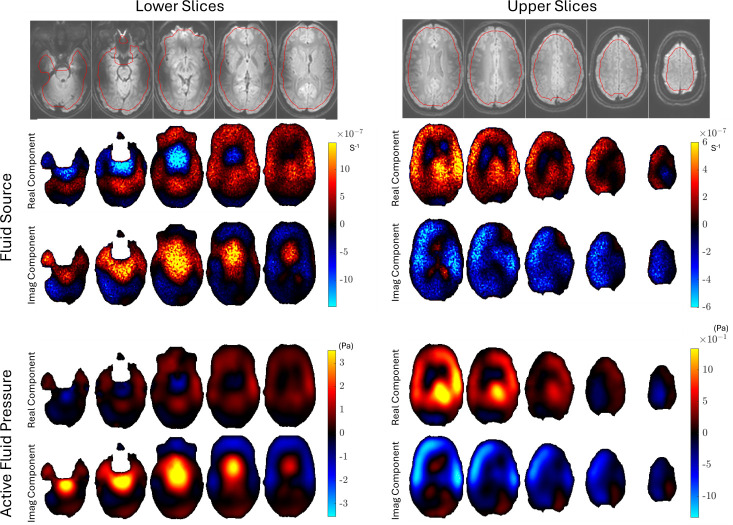
Example fluid source and pore fluid pressure images for repeat 1 of subject 1. Pore fluid pressure is the complex-valued fundamental mode of the Fourier transform of the active fluid compartment (a continuum representation of the pulsatile pressure over all vessel sizes) and is not directly comparable to blood pressure. The imaginary part of the complex-valued Fourier amplitude corresponds approximately to systole in these images. Distributions of fluid sources and pressures look similar as expected; forcing fluid in or out directly affects the fluid pressure.

**Figure 6.**
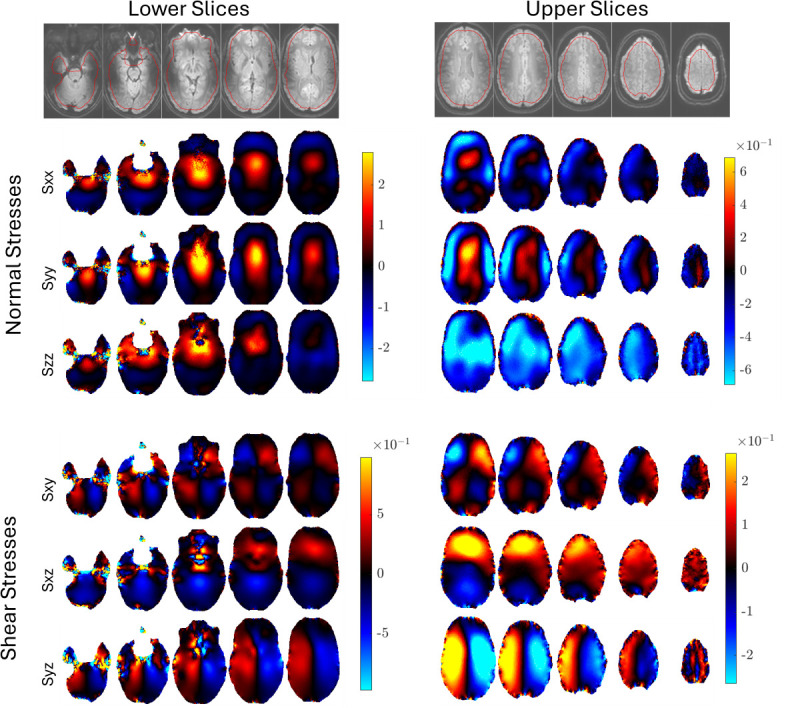
Example solid stress images for repeat 1 of subject 1. The imaginary component of the complex-valued Fourier amplitude is shown which corresponds approximately to peak systole. *x*, *y* and *z* in the stress components correspond to the AP, RL and FH directions, respectively. Units are Pa.

**Figure 7.**
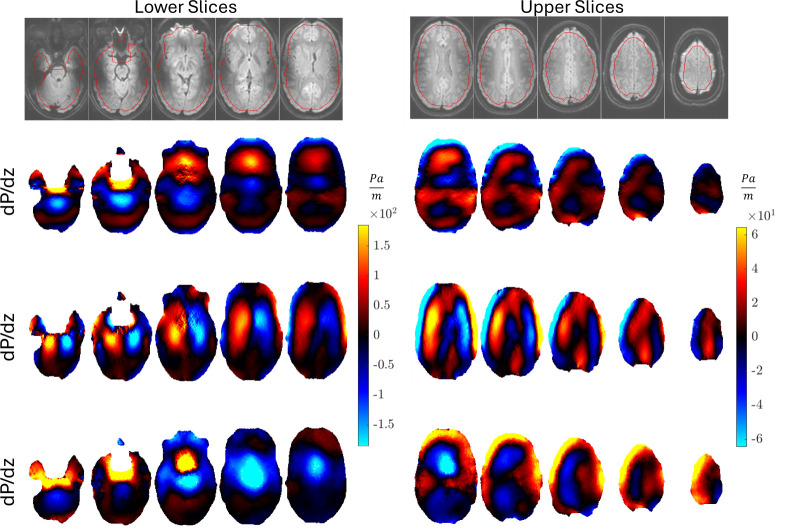
Example active fluid pressure gradient images for repeat 1 of subject 1. The imaginary component of the complex-valued Fourier amplitude is shown which corresponds approximately to peak systole. *x*, *y* and *z* in the gradient directions correspond to the AP, RL and FH directions, respectively.

Representative examples of the images produced by pgMRI are shown for repeat 1 of subject 1 in figures 4–7. Since our poroelastic model is constructed in the frequency domain under a time-harmonic steady-state cardiac pulsation assumption, all quantities of interest are complex-valued amplitudes, i.e. $f\left(x,t\right)=Re\left\{Fe^{i\omega t}\right\}.$ We show the fundamental mode of the Fourier transform in this initial study as the SNR is the highest, but similar images can be produced with higher harmonics of the cardiac waveform. Comparisons of displacement images from MRI measurements and the displacements from the fitted poroelastic model are shown in figure 4. The estimation process involves finding a fluid source, γ, which minimizes the GLS objective function equation (2.3) and yields a concomitant pressure field, P, both shown in figure 4. These displacements and pressures then provide the driving forces for the very slow pulsatile motion of ISF through the stresses in the porous solid matrix and fluid pressure gradients shown in figures 6 and 7.

**Figure 8.**
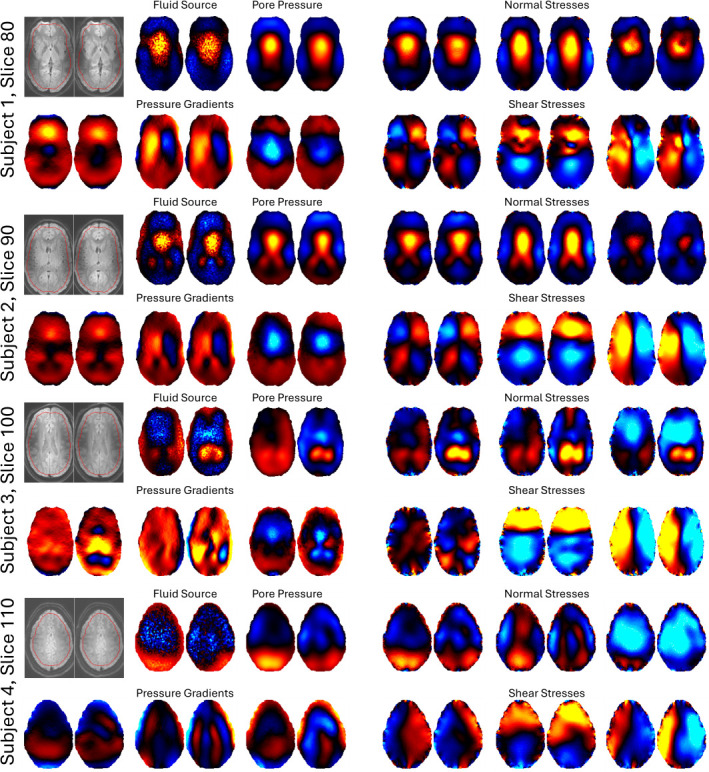
Repeatability of derived pgMRI images in four subjects. Image pairs show scan 1 and scan 2 for each subject (same colourmap scales). Left–right ordering of the stress components are normal stresses: $S_{xx}$, $S_{yy}$, $S_{zz}$; shear stresses: $S_{xy}$, $S_{xz}$, $S_{yz}$, and the pressure gradients are dP/dx, dP/dy, dP/dz, respectively. Display slices are deliberately chosen to be different for each subject to show multiple levels in the brain.

A demonstration of pgMRI repeatability for two scans of each subject (with repositioning) is shown in figure 8. The pgMRI parameter maps are quite repeatable, although subject 3 did show higher variability than the other participants. Global averages of each parameter type for the four subjects are presented in table 1, and the test–retest reliability is quantified by $\psi_{Q}=2\frac{\bar{|Q_{1}|}-\bar{|Q_{2}|}}{\bar{|Q_{1}|}+\bar{|Q_{2}|}}\times 100\%$, where the notation $\bar{|Q_{i}|}$ represents the global average of the magnitude of the arbitrary complex-valued parameter Q, for test i.

**Table 1.** Test–retest repeatability of pgMRI measures across four subjects during repeated scans with repositioning. The notation $\bar{\left|Q\right|}$ represents the global average of the magnitude of an arbitrary complex-valued quantity, Q. The test–retest performance is quantified by ψ, which is the ratio of the absolute difference and mean of the two tests, expressed as a percentage. Units are s^−1^ for the fluid source, γ; Pa for the pore pressure, P, and stress tensor components, $S_{ii}$; and Pa m^−1^ for the pressure gradients of the active fluid, $dP/dx_{i}$.

		subject 1			subject 2			subject 3			subject 4		
		Rep1	Rep2	ψ (%)	Rep1	Rep2	ψ (%)	Rep1	Rep2	ψ (%)	Rep1	Rep2	ψ (%)
** $\bar{\left|\gamma \right|}$ **		3.9 × 10^−7^	3.3 × 10^−7^	16	3.5 × 10^−7^	3.2 × 10^−7^	9	3.1 × 10^−7^	5.1 × 10^−7^	47	3.5 × 10^−7^	5.0 × 10^−7^	36
** $\bar{\left|P\right|}$ **		0.73	0.79	9	0.64	0.62	3	0.77	0.92	17	0.73	0.90	22
** $\bar{\left|S_{xx}\right|}$ **		0.46	0.44	4	0.39	0.38	3	0.58	0.59	0.8	0.48	0.53	11
** $\bar{\left|S_{yy}\right|}$ **		0.56	0.56	1	0.44	0.41	8	0.66	0.63	5.4	0.56	0.59	7
** $\bar{\left|S_{zz}\right|}$ **		0.65	0.60	8	0.59	0.54	10	0.83	0.84	0.6	0.67	0.72	8
** $\bar{\left|S_{xy}\right|}$ **		0.16	0.17	2	0.11	0.13	18	0.20	0.18	11.3	0.17	0.17	2
** $\bar{\left|S_{xz}\right|}$ **		0.22	0.23	2	0.17	0.19	10	0.30	0.26	14.1	0.26	0.26	1
** $\bar{\left|S_{yz}\right|}$ **		0.27	0.24	10	0.21	0.23	10	0.34	0.29	16.5	0.26	0.28	10
** $\bar{\left|dP/dx\right|}$ **		38.0	36.7	3	30.4	31.4	3	32.8	43.8	28.8	37.8	49.8	27
** $\bar{\left|dP/dy\right|}$ **		47.8	47.7	0	37.8	34.1	10	47.0	53.3	12.5	46.8	63.2	30
** $\bar{\left|dP/dz\right|}$ **		50.3	44.7	12	47.5	42.0	12	49.0	63.3	25.5	50.0	65.7	27

## 4. Discussion

pgMRI is a new technique which estimates the forces that drive pulsatile motion of ISF in living humans and has a range of potential clinical and basic science applications. The simulation shown in figure 3 demonstrates that the GLS fluid source recovery process is stable in the presence of levels of measurement noise higher than would be expected from *in vivo* measurements. The good match between the fitted displacements and measured data demonstrated in figure 4 across all displacement components suggests that a distributed fluid source is a good model for the source of cardiac-induced brain tissue pulsations. Artefacts or model–data mismatch in pure fluid spaces such the ventricles (we assume a homogeneous poroelastic material across the whole brain, including the ventricles) do not appear to cause major degradations in the fitting process.

The fluid source maps and/or pressure fields in figure 5 may be informative as they may elucidate disruptions in brain pulsatile behaviour. This possibility needs to be investigated further in future studies. A major advantage of assimilating measurements into a multi-parametric computational model is that we can use the data-driven model outputs to estimate otherwise unmeasurable quantities. Our simplified model of brain, conceptualized in figures 1 and 2, provides the stresses and fluid pressure gradients from the fitted poroelastic governing equations that presumably form the driving forces for very weak pulsatile ISF motion. The example fields in figures 6 and 7 show expected levels of symmetry and stretching in the LR and AP directions. Some expected anatomical details are not apparent in the images, primarily due to the homogeneous property assumptions applied in this preliminary work. The structure of the true brain stiffness distribution [39] will affect the stress fields, and spatial variations in hydrodynamic properties, such as the hydraulic conductivity [28] will influence the fluid source and pressure fields. Maps of these properties are available through IA-MRE or other methods. The homogeneous assumption was chosen to isolate the effects of the fluid and stress fields calculated by our new method and avoid potential confounding from errors introduced by other techniques. The movement of CSF may also contribute to artefacts in the pgMRI images. Future work will investigate whether accuracy is improved by including spatially varying mechanical and hydrodynamic property maps measured by other techniques, and whether CSF movement needs to be included in the model.

The repeatability of the pgMRI properties demonstrated in figure 8 and table 1 suggests that these new images of otherwise unmeasurable quantities can be reproduced consistently in the same subject. This finding gives confidence that perturbations in the measured fields due to diseases or other interventions will be detectable above the variability due to measurement system noise and normal physiological variations. Subject 3 had the lowest level of agreement (highest variability) between two repeated scans which may have resulted in subject movement introducing artefacts in the measured pulsatile displacement maps, or physiological variations from arterial blood flow changes due to blood pressure and heart rate differences during the scanning session, or changes in brain functional activity. Future work will investigate the imaging and physiological factors which affect pgMRI in more detail.

To our knowledge, no viable alternative methods are available to make these types of measurements for independent validation. Although two-photon microscopy has provided very important measurements of pulsatile ISF motion [10], this class of methods can examine only very small windows on the brain surface in rodents. More comprehensive validation in a larger population will be addressed in future work. The ability of pgMRI to detect relevant physiological changes will also be tested in 3D printed phantoms [40] and *in vivo* by perturbing the neurofluid pulsatile environment through controlled interventions and confirming pgMRI estimates change appropriately. Options include abdominal weights which increase venous return pressure and affect intracranial venous blood volume [41]. Interstitial pressure gradients increase as ICP increases; as intracranial compliance is reduced by pressurizing the abdomen, intracranial pressure gradients increase because ICP is coupled to venous pressure. Alternatively, hypercapnia which increases arterial blood volume due to higher CO_2_ levels [42–44] will increase intracranial pressure gradients.

Future work will optimize pgMRI parameters, such as the mechanical and hydrodynamical property maps (which can be prescribed in advance from literature values, or generated from patient-specific MRE property images). Initial experiments indicate that the pgMRI problem is not particularly sensitive to changes in assumed properties and BCs [32]; however, a more thorough investigation should be performed. We currently use the simplest model of uncorrelated fluid sources and measurement noise, which yields a diagonal structure for the weighting matrices $W_{\delta }$ and $W_{b}$. Expected levels of spatial correlation can be included in the fluid source maps through distance-based functions and the correlation of noise in the displacement measurements for each slice expected from MR sequences that assume steady-state motion over longer time periods [34] can be encoded in $W_{\delta }$.

The fluid source maps from pgMRI may also improve the accuracy of IA-MRE, which computes mechanical property maps from similar pulsatile displacement fields [17]. Currently, a subzone-based NLI algorithm [45,46] is used which assumes fluid sources are zero, and requires that the contribution from internal fluid sources is small relative to the strain provided by neighbouring subzones. Given that the brain is very well perfused throughout, this assumption contributes to the error of IA-MRE throughout the brain, and it is particularly problematic in subzones, which include large vessels where the pulsatile component of blood pressure is highest (such as around the circle of Willis [17]). A data-driven fluid source map could reduce error in mechanical property images in IA-MRE arising from this assumption [47], although the uniqueness of estimating both mechanical properties and fluid source maps from pulsatile data motion only would need to be investigated.

Finally, multicompartment poroelastic models may be applicable to estimating the actual ISF pulsatile fluid flow which results from the driving forces computed by pgMRI. Multicompartment models including venous blood, arterial blood, ISF and CSF components have been explored [16,48,49]. One or more of the pgMRI fluid source maps, stress fields and blood pressure gradients can be used to drive a multicompartment model, which will provide estimates of the pulsatile motion of any of the fluid compartments which may be of interest diagnostically (such as ISF). Multicompartment modelling might also be useful to confirm the validity of the assumption used in our current pgMRI derivation that the ISF pulsatility is very small relative to the pulsatility of the blood compartment, and therefore has a negligible effect on the macroscale brain motion.

Steady-state bulk flow of ISF caused by continuous turnover of fluid in the brain is also likely to be an important contributor to waste clearance in addition to the pulsatile effects measured here [7]. pgMRI does not estimate the steady-state ISF flow since the latter does not generate measurable displacements; however, other complementary techniques, such as contrast agent injection, have the potential to make these determinations, which potentially allows pulsatile and steady-state ISF to be investigated separately.

## 5. Conclusion

pgMRI is a novel data-driven, non-invasive method for estimating the forces which drive pulsatile ISF movement in living human brains and has the potential to become a relevant tool for clinical research. Simulations show that the fluid source recovery process presented here is stable in the face of measurement noise higher than levels expected from *in vivo* measurements. Recovered displacement maps from *in vivo* human data suggest that a distributed fluid source represents cardiac-induced brain tissue pulsations with reasonable accuracy, and further, model–data mismatch in pure fluid regions (e.g. in ventricles) does not cause major artefacts in the fitting process. Estimated solid stress and pressure gradient images demonstrate expected levels of symmetry and stretching in LR and AP directions, and repeatability studies of pgMRI properties indicate that these new images of otherwise unmeasurable quantities can be reproduced consistently in the same subject.

## Data Availability

Data and source code can be downloaded from https://doi.org/10.5061/dryad.4b8gthtq7 [50].
